# Endothelial injury in rheumatoid arthritis: a crosstalk between dimethylarginines and systemic inflammation

**DOI:** 10.1186/s13075-017-1232-1

**Published:** 2017-02-10

**Authors:** Theodoros Dimitroulas, James Hodson, Aamer Sandoo, Jacqueline Smith, George D. Kitas

**Affiliations:** 1 4th Department of Internal Medicine, Hippokration Hospital, School of Medicine, Aristotle University of Thessaloniki, Thessaloniki, Greece; 2 Department of Rheumatology, Russells Hall Hospital, Dudley Group NHS FT, Pensnett Road, DY1 2HQ Dudley, UK; 3 Institute of Translational Medicine, University Hospital Birmingham NHS Foundation Trust, Queen Elizabeth Hospital Birmingham, Mindelsohn Way, Birmingham, B15 2WB UK; 4 School of Sport, Health and Exercise Sciences, Bangor University, George Building, Bangor, Gwynedd, Wales LL57 2PZ UK; 5 Arthritis Research UK Epidemiology Unit, University of Manchester, Oxford Road, Manchester, M13 9PT UK

**Keywords:** ADMA, SDMA, Rheumatoid arthritis, cIMT, Inflammation

## Abstract

**Background:**

Symmetric (SDMA) and asymmetric (ADMA) dimethylarginines have emerged as novel biomarkers of cardiovascular disease (CVD) in several disease settings associated with atherosclerosis. Rheumatoid arthritis (RA) is a chronic systemic inflammatory disease characterized by high CVD mortality and morbidity. ADMA and SDMA levels are abnormal in RA patients, but their correlation with assessments of endothelial function and structure remains unknown. We aimed to investigate whether SDMA and ADMA are associated with carotid intima media thickness (cIMT) and arterial stiffness as well as non-invasive assessments of in vivo micro- and macrovascular endothelial function in RA patients with high systemic inflammatory load.

**Method:**

ADMA and SDMA levels were measured using immunoassays in 197 RA individuals. Twenty-six of these [23 (86.4%) females, median age 70, quartiles (60, 73)] were identified as having high inflammatory markers [erythrocyte sedimentation rate (ESR) >25 mm/hr and C-reactive protein (CRP) > 5 mg/L], and were compared to the remainder of the cohort. Patients underwent assessments of microvascular endothelium-dependent and endothelium-independent function [laser Doppler imaging with iontophoresis of acetylcholine (Ach) and sodium-nitroprusside (SNP) respectively], macrovascular endothelium-dependent and endothelium-independent function (flow-mediated dilatation and glyceryl-trinitrate-mediated dilation respectively), and vascular morphology [pulse wave analysis, and carotid intima media thickness (cIMT)].

**Results:**

Significant interactions with inflammation were detected in the associations between ACh and both SDMA (*p* = 0.014) and ADMA:SDMA ratio (*p* = 0.027), as well as between SNP and SDMA (*p* = 0.042) and between arterial stiffness and ADMA:SDMA (*p* = 0.036), with the associations being stronger in the patients with high inflammatory markers in each case.

**Conclusions:**

Besides their emerging role as markers of endothelial dysfunction SDMA and ADMA may promote endothelial injury in RA as mediators of the adverse effects of systemic inflammation on micro- and macrovasculature respectively in patients with active disease.

## Background

Patients with rheumatoid arthritis (RA) have an increased risk for cardiovascular disease (CVD), which appears to be of equal frequency and severity to those with diabetes mellitus of similar duration [1]. This elevated CVD risk is only partially explained by the higher prevalence of traditional CVD risk factors in RA [2] and accounts for almost 40–50% of all deaths resulting in premature mortality and reduced life expectancy amongst RA patients compared to the general population [3]. The precise pathogenic mechanisms of CVD remain partially understood. It appears that inflammation-induced accelerated atherosclerosis is the main contributor to the excess CVD risk in RA, as inflammatory processes in rheumatoid synovia and atherosclerotic plaque share striking similarities [4].

Derangement of endothelial function is a key early step in the initiation of vascular injury leading to accelerated atherosclerosis and the development of plaque. Nitric oxide (NO) is the main endothelium-derived regulator of vascular homeostasis with vasodilatory, anti-proliferative and anti-atherogenic properties [5]. Reduced NO synthesis and bioavailability can lead to endothelial dysfunction through several mechanisms such as loss of basal vasodilator tone, enhanced blood cell migration and raised local chemokine and/or cytokine release. In RA, systemic inflammation is a pivotal player in the disruption of the equilibrium between the production of NO and other vasoactive agents and the ensuing imbalance initiates platelet activation, abnormal fibrinolytic activity, lipid and glucose metabolism disorders as well as oxidative stress, all of which contribute to impaired vascular patency and a hypercoagulable state [6–8]. There is a continuous need for the investigation of potential pathogenetic pathways that may shed more light in the complex interrelations between vascular dysfunction and systemic inflammation in RA.

Recently, interest has shifted towards the identification of molecules associated with suppression of NO synthase activity, the enzyme responsible for NO synthesis. Asymmetric (ADMA) and symmetric (SDMA) dimethylarginines are analogues of L-arginine - the precursor of NO - naturally liberated in biological fluids following proteolysis. They inhibit NO synthesis by competing with L-arginine at the active site of NO synthase. Regulation of ADMA metabolism depends on the enzymatic activity of dimethylarginine dimethylaminohydrolase whilst SDMA is mainly cleaved by kidneys, with ADMA:SDMA ratio suggested as an index of dimethylarginine dimethylaminohydrolase activity [9]. Dimethylarginines have emerged as novel markers of endothelial dysfunction and CVD risk in several conditions associated with severe vascular disease, such as coronary artery disease [10], renal failure [11], diabetes mellitus [12] as well as in the general population [13]. A recent meta-analysis of 22 prospective studies suggested that ADMA is strongly associated with CVD outcomes in a broad range of circumstances [14], confirming previous reports and illustrating a role for dimethylarginines in the assessment of CVD risk in high-risk populations. In RA, we and others have demonstrated abnormal circulating levels of ADMA and SDMA and established robust correlations with other indices of atherosclerotic burden, namely insulin resistance and cumulative systemic inflammatory load [15–18]. In RA, ADMA has been associated with morphological markers of subclinical atherosclerosis, such as carotid intima-media thickness (cIMT) in some [19] but not all studies [15, 20]. Published data employing non-invasive techniques of endothelium-dependent and endothelium-independent peripheral micro- and macrovascular function and pulse wave analysis have failed to establish relationships between dimethylargines and measurements of vascular morphology [21]. However, most of these studies included unselected RA patients with a mixture of high, moderate, low and/or minimal disease activity. Although endothelial function and structure are affected in patients with RA [22], no clear association between vascular abnormalities and systemic inflammation has been demonstrated [23].

The aim of the current study was to address whether dimethylarginine levels correlate with structural and functional markers of atherosclerosis specifically in RA patients with high systemic inflammatory load.

## Methods

### Patient characteristics

One hundred and ninety-seven consecutive patients were recruited from the Dudley Rheumatoid Arthritis Co-morbidity Cohort (DRACCO), a prospective study examining CV burden in RA. Detailed characteristics of the participants have been described previously [17]. Exclusion criteria included confirmed acute coronary syndrome, evidence of chronic kidney disease or serious psychiatric disorders. The study received ethics approval from The Black Country Research Ethics Committee and all participants gave their written informed consent according to the Declaration of Helsinki. In the present study, RA patients with high inflammatory markers defined as erythrocyte sedimentation rate (ESR) > 25 mm/hr and C-reactive protein (CRP) > 5 mg/L were identified and analysed as a distinct group. Comparisons were performed between the two subgroups of RA patients: the one with high inflammatory markers and the remaining patients with normal values of ESR and CRP.

### Protocol

All patients reported to a thermoregulated (22 ± 0.9 °C) vascular laboratory after a 12-hour overnight fast. They were asked to refrain from exercise 24 hours before the session, and from smoking 12 hours before the session. Drug regimens were not interrupted. All participants underwent a detailed clinical examination and demographic information was collected by questionnaire. A blood sample was also obtained on the same day, for the assessments of routine hematologic and biochemistry, lipid profile, fasting glucose, fasting insulin, and acute phase response. ESR was measured using the Starrsed Auto Compact blood sedimentation instrument, which automatically carries out the erythrocyte sedimentation analysis according to the Westergren reference method. CRP was measured using the VITROS™ 5,1 FS Chemistry System (Ortho-Clinical Diagnostics, Raritan, NJ, USA). This is an immunoturbidimetric method. The antibody in the reagent combines with CRP in the specimen thereby increasing turbidity in the solution. This is measured as an increase in absorbance at 340 nm. The identification of patients with high/abnormal inflammatory markers (ESR > 25 mm/hr and CRP > 5 mg/L) was performed on the basis of the upper limits of normal as provided by the lab. All biochemical tests were carried out in the Biochemistry Laboratory at Russells Hall Hospital, The Dudley Group NHS Foundation Trust, UK.

#### Microvascular endothelial function

Endothelial function of the microvasculature was assessed non-invasively using laser Doppler imaging (LDI) (Moor laser Doppler imager, Moor Instruments Ltd, Axminster, UK) with iontophoresis of 1% acetylcholine (ACh, endothelium-dependent, Miochol-E, Novartis, Camberley, UK) and 1% sodium-nitroprusside (SNP, endothelium-independent, Nitroprussiat Fides, Rottapharm, Barcelona, Spain) in 2.5 ml solution containing 0.5% saline by a single observer (AS). This technique has an intra-observer co-efficient of variation for ACh and SNP of 6.5% and 5.9% respectively for AS [24].

#### Macrovascular endothelial function

Assessment of macrovascular endothelium-dependent function was performed using flow-mediated dilatation (FMD) with high-resolution ultrasonography of the brachial artery (Acuson Antares ultrasound system, Siemens PLC, Camberley, UK) according to previously established guidelines [25]. The intra-observer co-efficient of variation for the study ultrasonographer (AS) was 10.7% for flow-mediated dilatation (FMD) and 11.8% for glyceryl-trinitrate-mediated dilatation (GTN) assessments respectively. For all vascular tests, endothelial function was expressed as the percentage increase in perfusion or diameter from baseline, and all analysis was carried out offline by AS, who was blinded to the identity of the patient.

#### Carotid atherosclerosis

High-resolution ultrasonography of the carotid artery was performed by an experienced ultrasonographer (AS) according to previously established guidelines [26]. The cIMT was defined by determining the thickness between the lines of Pignoli; with the first echogenic line representing the lumen-intima interface, and the second line representing the media-adventitia interface [27]. Assessments of cIMT were performed in the far wall, 1 cm proximal to the carotid bulb, at sites free of plaque in both the right and left common carotid arteries, using the longitudinal scanning plane. Three measurements were taken on each side, and these were averaged to give the mean cIMT for the right and left carotid arteries separately. The cIMT from both sides were further averaged to give the overall cIMT. The intra-observer co-efficient of variation was 8.6%.

#### Pulse wave analysis

Pulse wave analysis (SphygmoCor Px Pulse Wave Analysis, ScanMed Medical Instruments, Moreton-in-Marsh, UK) was used to determine subendocardial viability ratio (SEVR) and augmentation index (AIx) as described previously [28]. Briefly, an applanation tonometer was positioned over the radial artery with enough pressure to flatten (but not occlude) the patient’s artery. The applanation tonometer detects the pulse pressure wave, which is calibrated against the standard brachial blood pressure and gives the maximum (systolic) and minimum (diastolic) points of the pressure wave. The pressure waveform is then mathematically transformed into a central aortic waveform, which contains the first (peak flow) and second (peak pressure) systolic peaks and displays information on ejection duration. SEVR is calculated by utilising the ejection duration, which is automatically measured by the Sphygmocor system, and dividing the area under the diastolic curve (coronary perfusion) by the area under the systolic curve (cardiac workload). In normal conditions, SEVR can be anywhere between 130% and 200%. Values that are below unity (100%) reflect poor perfusion of the subendocardium.

#### Measurement of ADMA and SDMA

ADMA levels were measured in serum samples by using a commercial enzyme immunoassay ELISA kit (Immundiagnostik, Bensheim, Germany) as previously described [17]. The intra-assay coefficient of variation was 7.6%.

The SDMA assay is based on the method of competitive enzyme-linked immunoassays. The sample preparation includes the addition of a derivatization reagent for SDMA coupling. During the incubation period, the target SDMA in the sample competes with the SDMA derivative (tracer) immobilised on the wall of the microtiter plates for the binding of the polyclonal antibodies. The SDMA in the sample displaces the antibodies out of the binding to the tracer. Therefore, the concentration of the tracer-bound antibody is inversely proportional to the SDMA concentration in the sample. The absorbance is measured at 450 nm and patient samples are read from a standard curve. The intra-assay standard deviation was 7.5% and inter-assay was 6%. The lowest amount detected was 0.05 μmol/L.

### Statistical analysis

Prior to the analysis, the distributions of all variables being considered were assessed. Log_2_-transfomations were applied to any variables found to be skewed, in order to normalise the distributions and improve model fit. For variables with negative values, a constant was added to all cases before the log_2_-transfomation, to ensure that this was calculable in all cases. There were several clear outliers in the SDMA measurements, which were excluded from the main analysis, in order to produce reliable models for the remaining cases.

Patients with high levels of inflammation were compared to the group with normal inflammatory markers, using *t* tests for normally distributed (or log-transformed) variables, Mann–Whitney tests for variables that were not normally distributed, and Fisher’s exact tests for dichotomous variables.

General linear models were then produced, in order to assess how the relationships between ADMA and/or SDMA and a range of factors differed by the degree of inflammation. A dichotomous variable indicating the inflammation group (normal or high) was added as an independent variable in each model, along with the factor being considered. An interaction term between these variables was also included, to test whether the gradient for the factor being considered differed between patients with normal and high levels of inflammation.

Where significant interactions were detected, the resulting models were plotted, in order to visualise how the relationships between ADMA and/or SDMA and the factor being considered differed by inflammation. The coefficients from these models were also anti-logged and converted into gradients, representing the percentage increase in ADMA and/or SDMA for a unit increase in the factor.

A sensitivity analysis was also performed, in order to assess whether excluding the outliers had biased the sample and influenced the conclusions. For the whole cohort, Spearman’s correlation coefficients between the factors and outcomes were produced separately for the high and normal inflammation groups. The resulting coefficients were then compared, to highlight any cases where the relationships between variables differed by the level of inflammation.

All analyses were performed using IBM SPSS Statistics 22 (IBM Corp. Armonk, NY, USA). Missing data were excluded on a per analysis basis and *p* < 0.05 was deemed to be indicative of statistical significance.

## Results

### Patients, characteristics, outliers

Data were available for 197 RA individuals. After being log_2_-transformed, SDMA was found to closely follow a normal distribution, with the exception of 11 (6%) patients with SDMA values >1. These outliers were excluded, in order to make parametric analysis valid, and to prevent them from becoming excessively influential in the analyses performed, leaving *N* = 186. Of these patients, 26 (14%) were identified as having high levels of inflammation, as defined above.

Comparisons between the high and normal inflammation groups are reported in Table 1. With the exception of disease activity score 28, which was found to be increased in the high inflammation group as expected, none of the other factors were found to differ significantly between the two groups. Table 2 reports the *p* values from the models used to test for interactions between inflammation and the factors being considered. Significant interactions with inflammation were detected in the associations between vasodilatory responses to acetylocholine (Ach) and both SDMA (*p* = 0.014) and ADMA:SDMA ratio (*p* = 0.027), as well as between vasodilatory responses to 1% sodium-nitroprusside (SNP) and SDMA (*p* = 0.042) and between augmentation index (AIx) and ADMA:SDMA ratio (*p* = 0.036). In each of these cases, it was found that the associations between the factors and SDMA or ADMA:SDMA ratio were stronger in the high inflammation group compared to the normal inflammation group (Table 3).

**Table 1:** Demographics and RA disease characteristics and surrogate markers of atherosclerosis and endothelial dysfunction in RA patients

Factor		All patients	Inflammation		
	*N*	(*N* = 186)	Normal (*N* = 160)	High (*N* = 26)	*p* value
Age (years)^b^	186	66 (59, 73)^b^	66 (58, 72)	70 (60, 73)	0.225
Female gender (%)^a^	186	144 (77.4%)	121 (75.6%)	23 (88.5%)	0.206
Disease duration (years)^b^	154	16 (11, 25)^b^	16 (11,25)	16 (12. 21)	0.692
RF (%)^a^	183	136 (74.3%)	114 (72.2%)	22 (88.0%)	0.137
Anti-CCP antibodies (%)^a^	181	115 (63.5%)	96 (61.5%)	19 (76.0%)	0.186
ESR (mm/h)^b^	181	11 (5, 22)^b^	9 (5, 16)	40 (30, 56)	-
CRP (mg/L)^b^	186	3.0 (2.9, 8.0)^b^	2.9 (2.9, 4.5)	21.0 (13.0, 31.0)	-
DAS 28^c^	181	3.14 (1.18)^c^	2.97 (1.10)	4.19 (1.12)	**<0.001**
ADMA (μmol/L)^d^	181	0.56 (0.54–0.58)^d^	0.55 (0.53–0.57)	0.59 (0.56–0.63)	0.136
SDMA (μmol/L)^d^	186	0.46 (0.44–0.47)^d^	0.46 (0.45–0.47)	0.45 (0.41–0.50)	0.812
ADMA:SDMA^d^	181	1.22 (1.17–1.27)^d^	1.20 (1.15–1.25)	1.30 (1.17–1.46)	0.167
Arterial stiffness^c^	165	33 (9)^c^	33 (9)	33 (8)	0.791
Microvascular endothelial dependent (ACh %)^d^	179	235 (207–265)^d^	244 (213–276)	184 (119–262)	0.145
Microvascular endothelial- independent (SNP %)^d^	179	116 (100–134)^d^	119 (102–138)	100 (60–159)	0.421
Macrovascular endothelial- dependent (FMD %)^d^	173	9.8 (8.8–10.8)^d^	9.9 (8.8–11.0)	9.0 (6.4–11.9)	0.554
Macrovascular endothelial- independent (GTN %)^d^	155	19.6 (18.2–21.1)^d^	19.7 (18.2–21.4)	18.9 (16.0–22.4)	0.715
cIMT (mm)^d^	157	0.68 (0.66–0.70)^d^	0.67 (0.65–0.70)	0.72 (0.67–0.79)	0.116

Bold *p* values are significant at *p* < 0.05

*RA* rheumatoid arthritis, *RF* rheumatoid factor, *anti-CCP* anti–citrullinated protein antibody, *ESR* erythrocyte sedimentation rate, *CRP* C-reactive protein, *DAS 28* disease activity score 28, *ADMA* asymmetric dimethylarginine, *SDMA* symmetric dimethylarginine, *ACh* acetylcholine, *SNP* sodium nitroprusside, *FMD* flow-mediated dilatation, *GTN* glyceryl-trinitrate mediated dilatation, *cIMT* intima media thickness

^a^ *N* (%), with *p* values from Fisher’s exact tests

^b^Median (quartiles), with *p* values from Mann–Whitney tests

^c^Mean (SD), with *p* values from *t* tests

^d^Geometric mean (95% CI), with *p* values from *t* tests on log-transformed data

**Table 2:** *p* values from general linear models

	ADMA			SDMA			ADMA:SDMA		
Factor	Main Eff.	Inflam.	Int.	Main Eff.	Inflam.	Int.	Main Eff.	Inflam.	Int.
Log_2_ACh	0.390	0.954	0.636	**0.008**	**0.030**	**0.014**	**0.007**	0.093	**0.027**
Log_2_SNP	0.702	0.989	0.824	0.074	0.056	**0.042**	0.100	0.151	0.087
Log_2_FMD	0.363	0.767	0.677	0.420	0.097	0.092	0.174	0.138	0.110
Log_2_GTN	0.754	0.702	0.823	0.799	0.812	0.868	0.959	0.607	0.737
Log_2_cIMT	0.217	0.323	0.051	0.550	0.265	0.562	0.607	0.953	0.259
Log_2_SEVR	0.876	0.727	0.706	**0.006**	**0.049**	0.052	0.058	0.252	0.268
AIx (HR 75)	0.204	0.367	0.246	0.205	0.221	0.127	0.050	0.097	**0.036**

*p* values from general linear models, with three terms included in each model: Main Eff. – the main effect of the factor being considered, Inflam. – a binary variable, stating whether a patient had high levels of inflammation (ESR > 25 and CRP > 5), Int. – an interaction term between Main Eff. and Inflam., testing whether the relationship between the factor and outcome differs by the degree of inflammation. Bold *p* values are significant at *p* < 0.05

*ADMA* asymmetric dimethylarginine, *SDMA* symmetric dimethylarginine, *ACh* acetylcholine, *SNP* sodium nitroprusside, *FMD* flow-mediated dilatation, *GTN* glyceryl-trinitrate mediated dilatation, *cIMT* intima media thickness, *SEVR* subendocardial viability ratio, *AIx* augmentation index, *HR* heart rate

**Table 3:** Results from general linear models for factors with significant interaction terms

		Normal inflammation		High inflammation	
Outcome	Factor	Gradient	*p* value	Gradient	*p* value
SDMA	Log_2_ACh	−0.7% (−5.6%, 4.5%)	0.791	−16.3% (−26.3%, −5.0%)	**0.006**
ADMA:SDMA	Log_2_ACh	2.2% (−4.5%, 9.4%)	0.528	25.6% (6.1%, 48.7%)	**0.008**
SDMA	Log_2_SNP	0.4% (−2.4%, 3.3%)	0.778	−6.2% (−11.5%, −0.5%)	**0.034**
ADMA:SDMA	AIx (HR 75)	−0.1% (−0.5%, 0.4%)	0.827	1.6% (0.1%, 3.1%)	**0.033**

Results are from the models reported in Table 2. Since the outcome variables of SDMA and ADMA:SDMA had been log-transformed prior to analysis, the resulting coefficients were anti-logged, and converted into gradients representing the percentage increase in the outcome for a one unit increase in the factor. For the log-transformed factors, this is equivalent to the change associated with a twofold increase in the untransformed factor. Bold *p* values are significant at *p* < 0.05

*SDMA* symmetric dimethylarginine, *ACh* acetylcholine, *ADMA* asymmetric dimethylarginine, *SNP* sodium nitroprusside, *AIx* augmentation index, *HR* heart rate

### Microvascular function

**Fig. 1:**
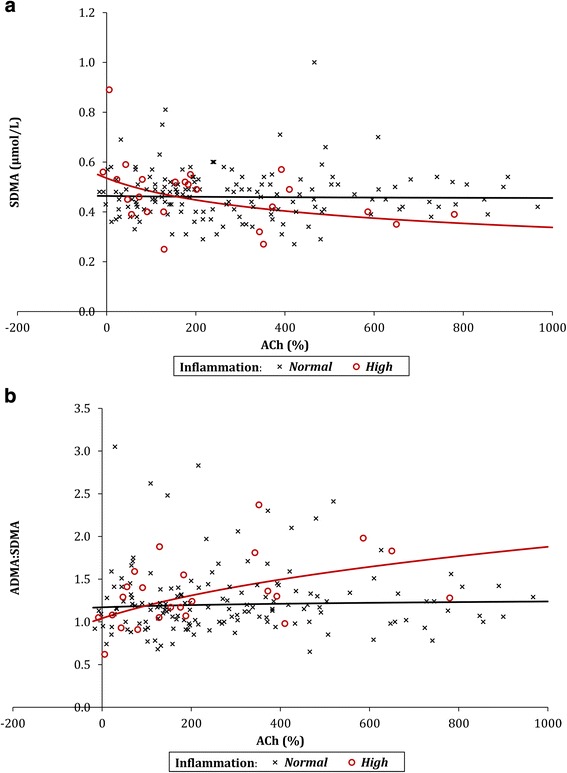
**a** The relationship between ACh and SDMA. **b** The relationship between ACh and ADMA:SDMA. *ACh* acetylcholine, *ADMA* asymmetric dimethylarginine, *SDMA* symmetric dimethylarginine

For those patients with normal inflammatory markers, there was no significant association between ACh and SDMA (*p* = 0.791). However, a significant negative correlation was detected in patients with high levels of inflammation (*p* = 0.006) (Fig. 1a). In this cohort, a twofold increase in ACh was found to be associated with a 16% reduction in SDMA.

The relationship between ACh and ADMA:SDMA was also non-significant in patients with normal levels of inflammation (*p* = 0.528). However, in patients with high levels of inflammation, a twofold increase in ACh was associated with a significant (*p* = 0.008) increase of 26% in ADMA:SDMA (Fig. 1b).

#### SNP

**Fig. 2:**
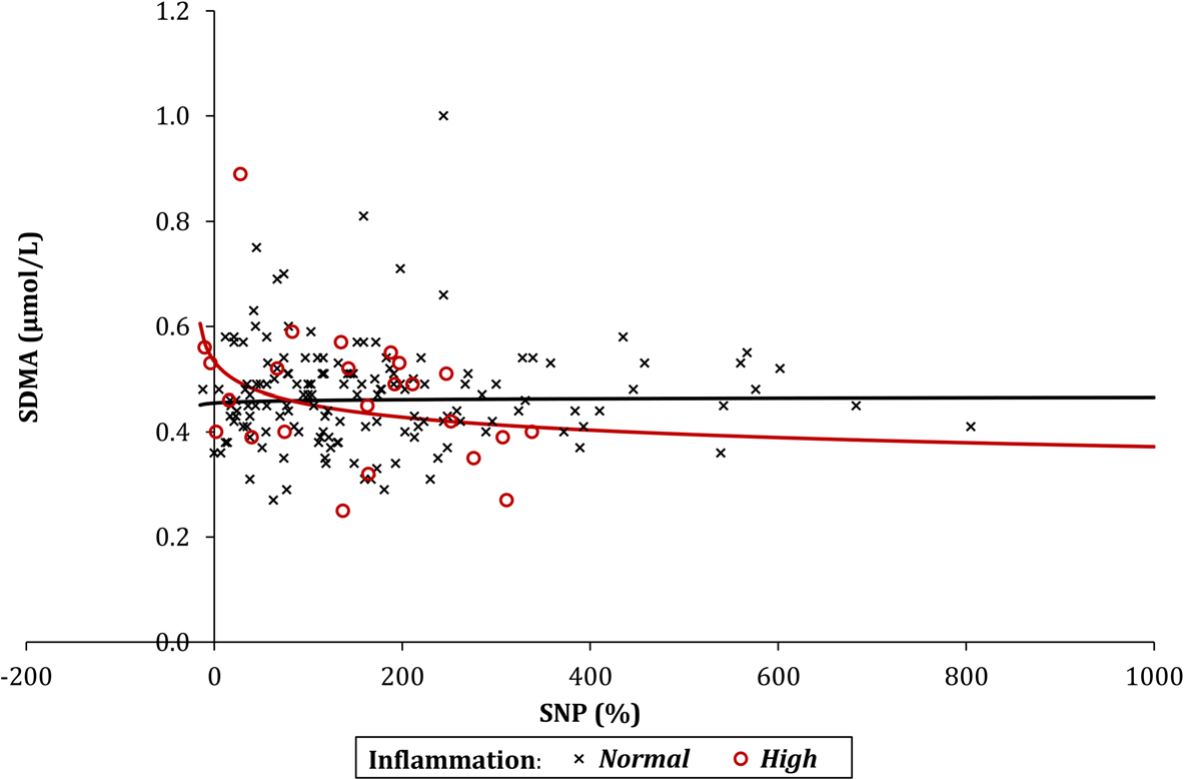
The relationship between SNP and SDMA. *SNP* sodium nitroprusside, *SDMA* symmetric dimethylarginine

No significant association between SNP and SDMA was detected in the group of patients with normal inflammation levels (*p* = 0.778). However, in the patients with high levels of inflammation, a significant negative correlation was detected (*p* = 0.034), with SDMA found to decline by 6.2% for each twofold increase in SNP (Fig. 2).

### Macrovascular function

No significant associations between dimethylarginines and assessments of macrovascular endothelial function were detected either in the group of patients with normal or in those with high levels of inflammatory markers.

### Arterial stiffness

**Fig. 3:**
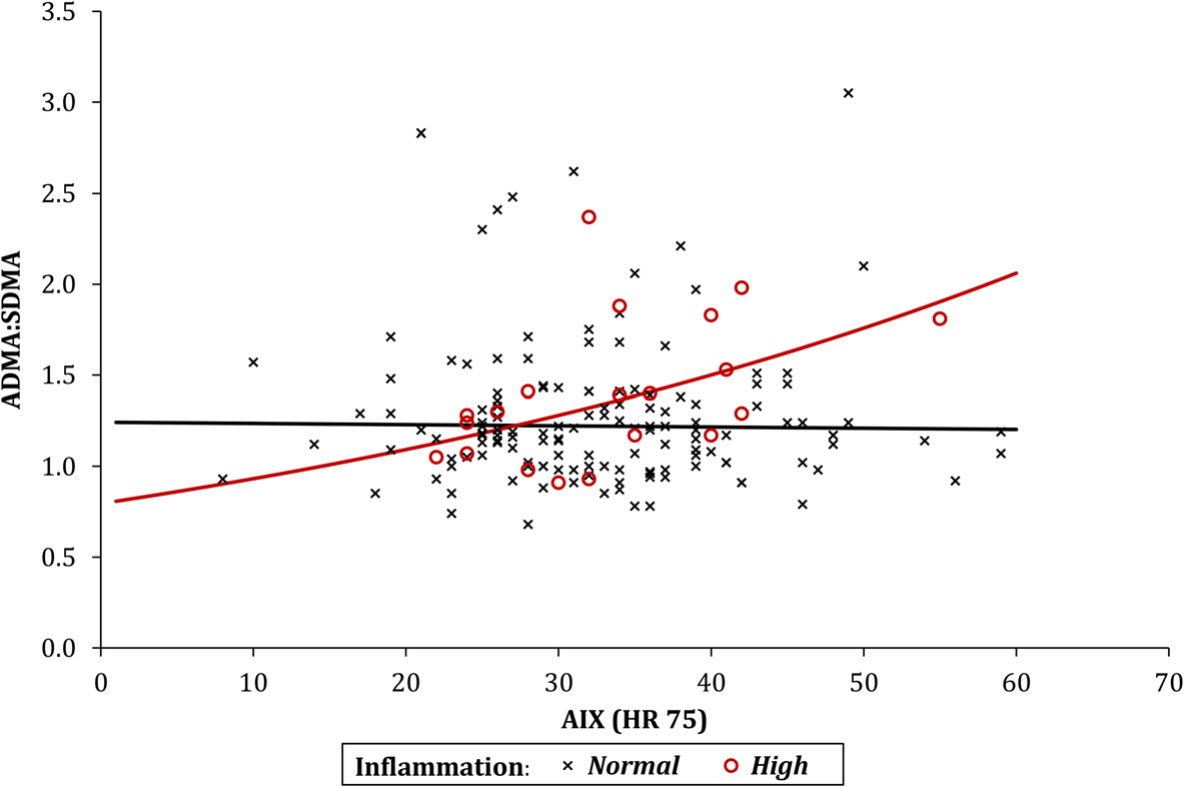
The relationship between AIx and ADMA:SDMA. *ADMA* asymmetric dimethylarginine, *AIx* augmentation index, *HR* heart rate, *SDMA* symmetric dimethylarginine

No significant association between AIX and ADMA:SDMA ratio was detected in the group of patients with normal inflammation levels (*p* = 0.827). However, in the patients with high levels of inflammation, a significant positive correlation was detected (*p* = 0.033), with ADMA:SDMA ratio found to increase by 1.6% for each unit increase in AIx (Fig. 3).

### Sensitivity analysis

A sensitivity analysis was performed on the whole cohort without exclusions (*N* = 197, 28 [14%] with high levels of inflammation). Since the outliers were now being included in the data, it was not possible to produce valid regression models, so a non-parametric approach was used. Patients were split into normal and high inflammation groups, and Spearman’s correlation coefficients produced to assess the level of correlation between variables (Table 4).

**Table 4:** Sensitivity analysis

	ADMA		SDMA		ADMA:SDMA	
Inflammation	Normal	High	Normal	High	Normal	High
Ach%	0.067 (*p* = 0.397)	0.251 (*p* = 0.216)	−0.077 (*p* = 0.325)	**−0.401 (** ***p*** **= 0.042)**	0.128 (*p* = 0.108)	**0.406 (** ***p*** **= 0.040)**
SNP%	0.016 (*p* = 0.840)	0.173 (*p* = 0.397)	−0.018 (*p* = 0.816)	**−0.404 (** ***p*** **= 0.040)**	0.023 (*p* = 0.776)	0.384 (*p* = 0.053)
FMD%	−0.002 (*p* = 0.976)	0.185 (*p* = 0.365)	0.099 (*p* = 0.220)	−0.165 (*p* = 0.421)	−0.076 (*p* = 0.353)	0.280 (*p* = 0.165)
GTN%	−0.023 (*p* = 0.782)	−0.090 (*p* = 0.698)	−0.052 (*p* = 0.540)	0.007 (*p* = 0.978)	0.033 (*p* = 0.698)	−0.090 (*p* = 0.697)
cIMT	0.100 (*p* = 0.247)	**−0.482 (** ***p*** **= 0.017)**	0.005 (*p* = 0.958)	−0.231 (*p* = 0.278)	0.064 (*p* = 0.466)	−0.101 (*p* = 0.637)
SEVR	−0.013 (*p* = 0.869)	0.039 (*p* = 0.865)	0.124 (*p* = 0.121)	0.255 (*p* = 0.251)	−0.091 (*p* = 0.264)	−0.303 (*p* = 0.170)
AIx (HR75)	−0.011 (*p* = 0.893)	0.272 (*p* = 0.222)	0.069 (*p* = 0.398)	−0.287 (*p* = 0.195)	−0.065 (*p* = 0.434)	**0.527 (** ***p*** **= 0.012)**

Values are quoted as Spearman’s correlation coefficients with *p* values. Bold coefficients/*p* values are significant at *p* < 0.05

*ADMA* asymmetric dimethylarginine, *SDMA* symmetric dimethylarginine, *ACh* acetylcholine, *SNP* sodium nitroprusside, *FMD* flow-mediated dilatation, *GTN* glyceryl-trinitrate-mediated dilatation, *cIMT* intima media thickness, *SEVR* subendocardial viability ratio, *AIx* augmentation index, *HR* heart rate

The results were consistent with the main analysis, with significant negative correlations detected between SDMA and both ACh and SNP in patients with high inflammation that were not observed in those with normal inflammation. ADMA:SDMA ratio was found to be significantly positively correlated with ACh and AIx in patients with high inflammation, but not in those with normal inflammation, as in the main analysis. The sensitivity analysis additionally identified a significant negative correlation between cIMT and ADMA in patients with high inflammation that was not present in those with normal inflammation. This effect was not found to be significant in the main analysis, although it only narrowly missed significance, with *p* = 0.051.

## Discussion

The main finding of our study is the establishment of statistically significant relationships between circulating dimethylargines and assessments of microvascular function and arterial stiffness in RA patients with high levels of systemic inflammatory activity. To the best of our knowledge, this is the first time that such associations are reported in this population and our results may provide further insights in the complex interrelations between functional vascular changes, atherosclerosis and inflammation in RA.

Several non-invasive techniques for the assessment of endothelial morphology and function in micro- and macrovasculature can examine the development of premature atherosclerosis and provide useful information regarding an individual’s CVD risk status, particularly in clinical settings associated with excess CVD risk, such as RA [29]. In this study, we utilized a broad range of assessments to ensure the global evaluation of vascular function and morphology and investigate potential relationships between indices of vascular health and surrogate markers of endothelial dysfunction and systemic inflammation.

Our findings suggest that dimethylarginines represent not only biomarkers of endothelial dysfunction, but may themselves be important mediators of disruption of vascular homeostasis in RA. Besides their inhibitory properties on NO synthesis, dysregulated ADMA and SDMA metabolism may affect endothelial function through other mechanisms independent of NO synthase inhibition such as inflammation [30]. Under these circumstances, dimethylarginines contribute to the precipitation of oxidative stress by altering endothelial NO synthase activity, switching it to a superoxide synthase [31, 32] and acting as amplifiers, promoting vascular injury. This is in line with previous observations, demonstrating links between derangement of NO/ADMA pathway, oxidative stress and inflammation in RA individuals [17, 33, 34].

The inverse correlation between SDMA and microvascular function demonstrated in our study may look at first glance fundamentally counterintuitive. However, SDMA levels are lower in RA subjects compared to controls [16] similarly to what occurs in diabetes mellitus and insulin-resistant patients [35, 36]. It appears that common mechanisms affecting intracellular migration of endogenous dimethylarginines in RA and insulin resistance are associated with atherosclerosis in both conditions [1]. Given that SDMA competitively inhibits ADMA for entry into the cells, increased SDMA cellular uptake induced by decreased insulin sensitivity [37] indirectly contributes to raised serum ADMA levels and progression of vascular disease. This intriguing interaction could explain how lower SDMA levels are associated with microvascular endothelium dysfunction. Finally, the establishment of associations with both endothelium-dependent and endothelium-independent microvascular function suggests that particularly SDMA induces NO depletion through NO synthase-dependent and NO synthase -independent pathways.

Cross-sectional studies have indicated a possible time lag between endothelial injury and structural wall damage, as impairment of endothelial function occurs differently and in different vascular beds with microvascular function affected earlier than the macrovascular one in RA individuals [38–40]. Despite the cross-sectional design of these studies, which cannot confirm temporal relationships, it can be speculated that the reported associations between SDMA only with microvascular function may underscore the role of dimethylarginines in initiating endothelial damage under conditions where high systemic inflammatory markers are present. On the other hand, the correlations between ADMA and cIMT indicate a role for endogenous NO synthase inhibitors in the development of later stages of atherosclerosis in individuals with high levels of systemic inflammation, which concurs with reports linking elevated CRP with cIMT in RA population [41].

It is worth noting that, in the modern era, ‘Treat to Target’ strategies and biologic regimens have considerably reduced the proportion of patients with high disease activity, to the point that only about 10% of the individuals in our cohort presented with high levels of systemic inflammation and only seven with disease activity score 28 > 5.1. The results of our study indicate that subpopulations analysis may unfold mechanisms more prominent in specific subgroups of patients, for example those with high systemic inflammation. Once more, these findings confirm the effect of active disease on vasculature and emphasize the need for more aggressive management of CVD risk in this subpopulation. The clinical relevance of interventions specifically targeting ADMA- and SDMA-related pathways in RA patients with high inflammatory activity remains to be evaluated in future large studies focusing on specific end points. A recent double-blind randomized study demonstrated that supplementation of oral tetrahydrobiopterin - a cofactor for the production of NO downregulated by oxidative stress - improved FMD in a small cohort of RA patients [42], providing the rationale for further research.

The strengths and limitations of our study merit consideration. Since the number of patients with high inflammation was small, the analyses performed had relatively low statistical power, particularly for the interaction term in the model, which was the main focus of the study. As a result, small but genuine differences between the two groups may have been missed. For these reasons, it would be preferable to validate the findings from this analysis in an external cohort, in order to ensure reliability of the conclusions. The other limitation was the requirement to exclude those patients with high SDMA levels from the main analysis. Leaving these cases in the data meant that they became influential outliers in the models produced, resulting in poor model fit and breaking the assumption of normally distributed residuals. However, the sensitivity analysis performed on the data returned consistent results, implying that any bias introduced by excluding these patients had not fundamentally affected the conclusions of the study. Nevertheless, our study contains adequate functional assessments and morphological measurements of vasculature in a real-life population representative of ‘average’ RA patients attending routine rheumatology clinics.

## Conclusions

In conclusion, we demonstrated that microvascular function, arterial stiffness and cIMT are associated with circulating ADMA and SDMA levels in RA patients with high inflammatory markers. Although CVD represents an unresolved issue throughout the whole RA population, it appears that high systemic uncontrolled inflammatory activity poses the higher risk for CVD events in RA subjects in the short and long term. In that respect, the present study suggests a possible pathway through which high systemic inflammatory load exerts its deleterious effects on vascular wall by precipitating the interaction between dimethylarginines, and endothelial cells and diminishing NO production directly or indirectly. Dimethylarginines therefore, can be considered important mediators of endothelial dysfunction contributing to the oxidative stress burden in the vasculature and the development of CVD in this population.

## Abbreviations

ACh: Acetylcholine
ADMA: Asymmetic dimethylarginine
AIx: Augmentation index
cIMT: Carotid intima media thickness
CRP: C-reactive protein
CVD: cardiovascular disease
ESR: Erythrocyte sedimentation rate
FMD: Flow-mediated dilatation
NO: Nitric oxide
RA: Rheumatoid arthritis
SDMA: -Symmetric dimethylarginine
SEVR: Subendocardial viability ratio
SNP: 1% sodium-nitroprusside
